# Single-Center Retrospective Analysis of Neutrophil, Monocyte, and Platelet to Lymphocyte Ratios as Predictors of Complicated Appendicitis

**DOI:** 10.7759/cureus.29177

**Published:** 2022-09-14

**Authors:** Sattam A Halaseh, Marcos Kostalas, Charles A Kopec, Abdullah Nimer

**Affiliations:** 1 General and Colorectal Surgery, Torbay and South Devon National Health Service (NHS) Foundation Trust, Torquay, GBR; 2 Upper Gastrointestinal Surgery, Torbay and South Devon National Health Service (NHS) Foundation Trust, Torquay, GBR; 3 Faculty of Medicine, The University of Jordan, Amman, JOR

**Keywords:** surgical diagnosis, monocyte to lymphocyte ratio, platelet to lymphocyte ratio, neutrophil to lymphocyte ratio, complicated appendicitis, diagnosis of acute appendicitis, surgical acute abdomen

## Abstract

Aim

We look at the ability of the neutrophil-to-lymphocyte ratio (NLR), platelet-to-lymphocyte ratio (PLR), and monocyte-to-lymphocyte ratio (MLR) to differentiate between uncomplicated and complicated appendicitis.

Methods and materials

This was a retrospective, single-center study of 234 individuals diagnosed with acute appendicitis between January 1, 2020, and December 31, 2020. Patients were grouped into uncomplicated and complicated appendicitis subgroups. Patients with histologically or radiologically proven gangrenous or perforated appendicitis, as well as those with peritonitis or peri-appendiceal abscess development, comprise the complicated subgroup. Independent Mann-Whitney samples The U test was used to predict lab values of complicated appendicitis. Furthermore, the receiver operating characteristic (ROC) curve and area under the curve (AUC) were utilized to predict the sensitivity and specificity of laboratory results reported to have a significant connection with complex appendicitis

Results

The criteria were met by 186 patients, with a male-to-female proportion of 1.06:1, an average age of 36.4 years, and an average stay of 2.73 days. There were 95 individuals with complicated appendicitis. With 66.3%, perforated appendicitis was the most prevalent condition. The ratios of neutrophils to lymphocytes, monocytes to lymphocytes, and platelets to lymphocytes were linked with complicated appendicitis with a p-value of < 0.0001, and p-values = 0.015, and 0.015, respectively.

Conclusion

NLR, MLR, and PLR are valid, less onerous surrogate biomarkers for measuring the severity of acute complicated appendicitis and differentiating it from uncomplicated appendicitis.

## Introduction

Acute appendicitis represents one of the most prevalent causes of acute abdomen and is the major cause of emergency abdominal surgery. There are well-recognized typical symptoms and clinical features [1]; nevertheless, prompt diagnosis is not always easy [2] as one-third of patients lack these common symptoms [1]. The diagnosis of appendicitis is often made clinically however radiological investigations also aid the diagnosis. Perforation, which can be linked with considerable morbidity [3] and even fatality [4], is one potential complication of delayed diagnosis of acute appendicitis.

In developed countries, the incidence rate of acute appendicitis ranges between 5.7 and 50 individuals per 100,000 people per year, with a peak incidence between the ages of 10 and 30 [2]. It is 1.4 times more prevalent in men [5], but women are twice as likely to undergo an appendectomy [6]. The lifetime risk of acute appendicitis is estimated to be approximately 7%, with perforation rates reaching 20% of cases [5]. This condition's mortality risk is less than 1% among the general population, but it can reach 50% in the elderly population [5].

Laparoscopic appendicectomy is generally considered to be a safe procedure; yet, problems may emerge; therefore, it is crucial to minimize the likelihood of undergoing unnecessary appendectomies. Rapid and precise diagnosis has been shown to decrease the number of unnecessary appendicectomy procedures. Clinical symptom evaluation, scoring systems like the Alvarado score [7], and imaging techniques like ultrasonography and computed tomography (CT) scans are all helpful diagnostic tools for acute appendicitis. Acute appendicitis may be predicted objectively with the help of scoring systems; however, these methods are not very sensitive or specific and cannot reveal how far along the inflammatory process the patient is in [7]. CT scans are costly and not accessible in many hospitals, despite the fact that they can cut the likelihood of negative appendicectomy in half [5].

Therefore, it is of utmost importance to adopt low-cost, basic diagnostic methods in identifying acute appendicitis and minimizing the overall number of negative appendicectomies. The patient's hemoglobin levels, total leucocyte count, platelet count, and differential counts may all be determined with a simple, inexpensive, and easily accessible complete blood count. In spite of the fact that leucocytosis is a common finding in patients with acute appendicitis, its specificity is low because of the wide variety of abdominal illnesses that might cause it [7].

In multiple benign and malignant pathologies, including acute pancreatitis, coronary heart disease, oesophageal cancer, and colorectal cancer, the neutrophil-lymphocyte ratio (NLR) has been shown to be a more reliable predictor of unfavorable outcomes than the white blood cell count [8-11]. Moreover, hematologic component analyses in appendicitis have shown diverse immunological response patterns, with monocytes and neutrophils upregulated in complex appendicitis and basophils and eosinophils in non-complicated appendicitis [12,13]. Therefore, the neutrophil-to-lymphocyte ratio (NLR), which may be obtained from the differential count, can serve as an adjuvant in the diagnosis of acute appendicitis in general and aim to lessen the rate of negative appendicectomy. Consequently, the primary objective of our investigation was to identify potentially unique hematologic subpopulations for complex and uncomplicated appendicitis in our patients.

## Materials and methods

This is a retrospective, single-center analysis of all patients diagnosed with acute appendicitis conducted between January 1st, 2020, and December 31st, 2020. An institutional database was utilized to collect and compile patient information into a single spreadsheet. All patients who underwent either open or laparoscopic appendicectomy were included in the initial data set. For each participant in the study, the admission documentation, operation note, discharge summary, blood results, and histological findings were evaluated.

Notably, the blood tests reviewed were each patient's admission blood tests. Our patients are either presented to our emergency unit or surgical receiving unit. In each of these instances, blood tests are obtained promptly as part of the preliminary evaluation.

Inclusion criteria

**Figure 1:**
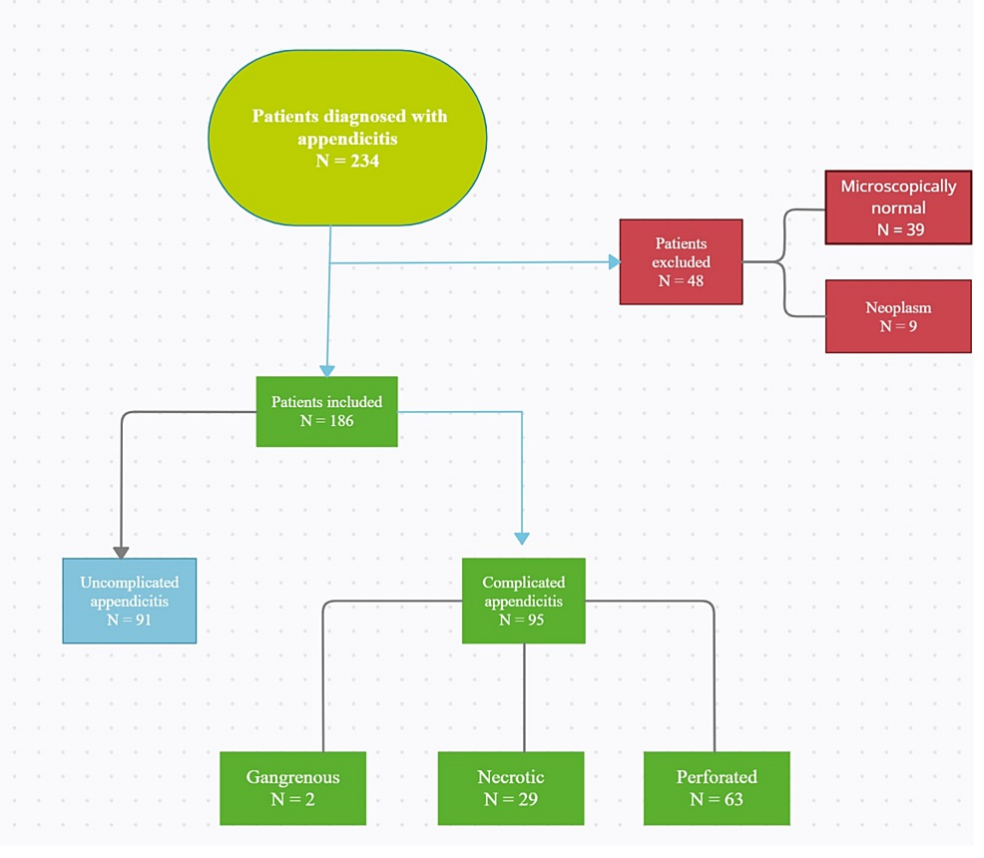
A flow chart represents the patient selection criteria.

The study included 234 patients who presented to Torbay General Hospital with acute appendicitis. Inclusion criteria were patients who had histopathological findings, CT scan findings, or ultrasound findings of acute appendicitis. The exclusion criteria were patients who had clinical suspicion of acute appendicitis but didn’t have any findings on histopathology (39 patients) and patients who had evidence of neoplastic findings on histopathology (nine patients). After applying the criteria, 186 patients were included in the quantitative analysis. Please refer to Figure 1 for patient selection criteria.

Definition

The patients were then categorized as complicated and uncomplicated appendicitis. The accepted definition of uncomplicated was the presence of histological or radiological evidence of simple, confined, or suppurative appendicitis. The complicated group includes patients with histologically or radiologically confirmed gangrenous or perforated appendicitis, as well as those with peritonitis or peri-appendiceal abscess formation. 

Statistical analysis

The patients’ data were entered using Microsoft Office Excel 2019, then imported into SPSS Statistics for Windows, Version 26.0 (IBM Corp., Armonk, NY) which was used to analyze the data. Simple counts and percentages were used for descriptive statistics along with the Chi-square test to show differences in means. Independent-Samples Mann-Whitney U test was used for lab values prediction of complicated appendicitis. Finally, the receiver operating characteristic (ROC) curve and area under the curve (AUC) was used to predict the sensitivity and specificity of lab values that were found to have a significant association with complicated appendicitis. For all tests, a confidence level of 95% was used and a p-value of <0.05 was significant.

## Results

Between January 1st, 2020, and December 31st, 2020, a total of 234 patients underwent an appendicectomy at our center. After applying the exclusion criteria as described above, a total of 186 patients were included in the quantitative analysis. Most of the sample were males (51.6%) and adults (76.3%). Ninety-five patients (51%) had complicated appendicitis, 57.9% of them were males and 75.8% of them were adults but the Chi-square test didn’t show statistical significance in the difference of means between groups. The average age of the cohort was 36.4 years. The most common approach was laparoscopic (86% of cases) and the average length of stay was 2.73 days. Table 1 describes the characteristics of the included patients.

**Table 1:** The characteristics of the included patients

		Total	Gender		Age group	
			Male	Female	Pediatrics	Adults
Total		186	96 (51.6)	90 (48.4)	44 (23.7)	142 (76.3)
Complicated appendicitis	Yes	95	55 (57.9)	40 (42.1)	23 (24.2)	72 (75.8)
	No	91	41 (45.1)	50 (54.9)	21 (23.1)	70 (76.9)
	P-Value		0.080		0.856	
Approach	Lap	160	77 (48.1)	83 (51.9)	36 (22.5)	124 (77.5)
	Open	16	7 (43.8)	9 (56.3)	6 (37.5)	10 (62.5)
	Converted	6	4 (66.7)	2 (33.3)	1 (16.7)	5 (83.3)
	Conservative	4	2 (50)	2 (50)	1 (25)	3 (75)
Average length of stay		2.73	2.66	2.81	2.52	2.80
	P-Value		0.200		0.746	
Average age		36.4	33.1	39.9	12.1	43.9

Perforated appendicitis accounted for 66.3% of the complicated appendicitis cases, followed by necrotic appendicitis (30.5%) and gangrenous appendicitis (2.1%). The incidence of complicated appendicitis in the cohort group is presented in Table 2.

**Table 2:** The complicated appendicitis types, and incidence among the cohort population.

Type of complication	Total	Gender		Age group		Average length of stay
	95	Male	Female	Pediatrics	Adults	
Gangrenous	2 (2.1)	1	1	1	1	5
Necrotic	29 (30.5)	19	10	9	20	4.31
Perforated	63 (66.3)	35	29	13	51	3.13

Initially, we investigated the full blood counts of patients with complicated appendicitis in greater detail. Each of these individuals was evaluated for white cell count (WCC), neutrophils, lymphocytes, monocyte, eosinophils, platelets, and red blood cell distribution width (RDW). We analyzed each of the WCC, neutrophil, and lymphocyte counts. None of the patients’ characteristics or RDW, neutrophil, monocyte, eosinophils, ELR, and platelets count was significantly associated with the occurrence of complicated appendicitis. There was a significant difference in WCC, red blood cell distribution to lymphocyte ratio (RDWLR), lymphocyte, monocyte to lymphocyte ratio (MLR), platelet to lymphocyte ratio (PLR), and neutrophil to lymphocyte ratio (NLR) between patients who had complicated vs. uncomplicated appendicitis as patients who had complicated appendicitis had significantly higher values. The results are shown in Table 3 below.

**Table 3:** Independent-Samples Mann-Whitney U Test showing the difference in lab value means between complicated and uncomplicated appendicitis cases. WCC: white cell count, RDW: red blood cell distribution width, RDWLR: red blood cell distribution width-to-lymphocyte ratio, NLR: Neutrophil-to-lymphocyte ratio, MLR: Monocyte-to-lymphocyte ratio, ELR: Eosinophil-to-lymphocyte ratio, PLR: Platelet-to-lymphocyte ratio.

	Complicated mean value	Uncomplicated mean value	Total mean value	P-Value
WCC (10*9/L)	14.15	12.88	13.53	.017
RDW (%)	13.79	13.35	13.58	.069
RDWLR	13.53	10.00	11.80	.005
Neutrophil (10*9/L)	11.63	10.19	10.92	.006
Lymphocyte (10*9/L)	1.35	1.57	1.46	.018
NLR	10.64	7.89	9.30	.000
Monocyte (10*9/L)	0.95	0.93	0.94	.730
MLR	0.79	0.68	0.74	.015
Eosinophils (10*9/L)	0.15	0.16	0.15	.266
ELR	0.12	0.10	0.11	.367
Platelet (10*9/L)	257.07	258.60	257.82	.724
PLR	247.17	190.54	219.46	.015

**Figure 2:**
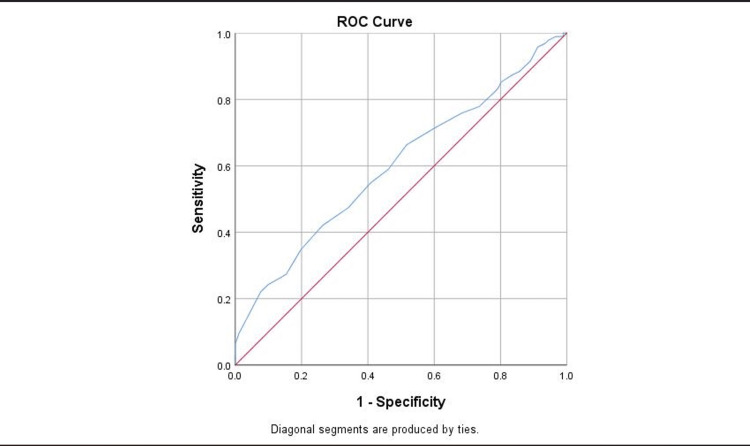
Receiver operating characteristics (ROC) curves of the lymphocyte.

**Figure 3:**
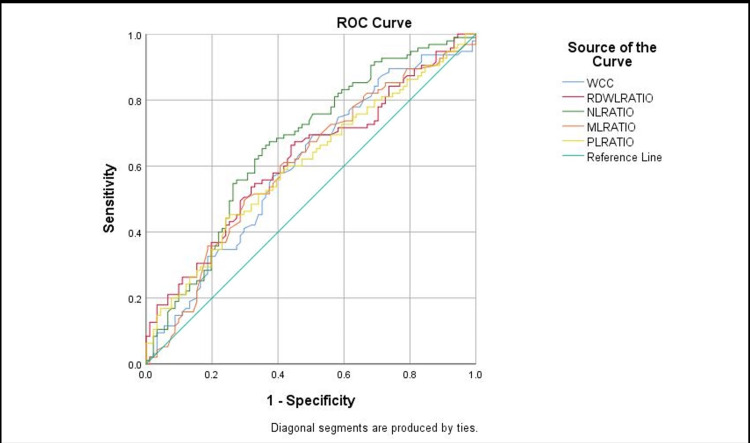
Receiver operating characteristics (ROC) curves of the WCC, MLR, RDWLR, PLR, and NLR. WCC: white cell count, RDWLR: red blood cell distribution width-to-lymphocyte ratio, NLR: Neutrophil-to-lymphocyte ratio, MLR: Monocyte-to-lymphocyte ratio, PLR: Platelet-to-lymphocyte ratio.

Sensitivity, specificity, and accuracy of the WCC, lymphocyte, MLR, RDWLR, PLR, and NLR were assessed. Using receiver operating characteristics (ROC) curves, the threshold value of the above components for diagnosing acute appendicitis and separating simple appendicitis from complex ones was identified (Figures 2, 3)

NLR with a cut-off value of 7.7 showed the highest accuracy with 67% (P <0.0001) compare to the other tests. Also, WCC with a cut-off value of 12.5 has the highest sensitivity with 69% vs. 67% compared to NLR. The results showed in Table 4 below.

**Table 4:** The Sensitivity, specificity, and accuracy of the WCC, lymphocyte, MLR, RDWLR, PLR, and NLR. WCC: white cell count, RDWLR: red blood cell distribution width-to-lymphocyte ratio, NLR: Neutrophil-to-lymphocyte ratio, MLR: Monocyte-to-lymphocyte ratio, PLR: Platelet-to-lymphocyte ratio.

Variable	AUC	P value	95% CI lower bound	95% CI upper bound	Suggested Cut-off value	Sensitivity	Specificity
NLR	0.67	<0.001	0.59	0.74	7.7	67%	63%
WCC	.610	.012	.527	.693	12.5	69%	52%
RDWLR	.624	.005	.542	.705	9	62%	56%
MLR	0.61	0.015	0.52	0.69	0.7	60%	60%
PLR	0.61	0.015	0.52	0.69	182	60%	53%
Lymphocytes (negative)	0.60	0.018	0.52	0.68	1.5	66%	54%

## Discussion

It might be difficult to recognize the symptoms of acute appendicitis in a timely manner, which can lead to needless complications and even death [14]. A surgeon has a difficult choice between waiting to operate until a full diagnosis can be made and operating early to avoid complications like perforation and peritonitis [15]. Although ultrasound and computed tomography (CT) scans are useful in making a diagnosis of appendicitis [16], not all institutions, especially those in rural areas, have access to these diagnostic tools. For this reason, NLR, or another simple, cheap, and straightforward diagnostic tool to aid and help surgeons detect cases of acute appendicitis, is essential.

Our research suggests that NLR should be considered for inclusion in a diagnostic tool for suspected patients of complicated appendicitis. While we acknowledge that validating such a screening tool is a priority, we believe it has the potential to be a beneficial development, particularly in light of the rising demands placed on healthcare systems worldwide. According to the results of this study, 51% of the patients who were diagnosed with acute appendicitis at our institution were complicated. Overall, male patients constituted 57.9% of the overall sample, and their average hospital stay lasted 2.66 days. The main etiology of complicated appendicitis was perforation with 66.3%.

When comparing patients with acute appendicitis with and without complications, there was a statistically significant difference in the mean of complete blood count components. WCC, RDW, neutrophil, and monocyte counts were all higher in the complicated appendicitis class, whereas lymphocyte and platelet counts were higher in the uncomplicated cluster. Individuals with complicated appendicitis, who were thought to have a more severe case of the condition, showed high levels of white blood cells (WBC) and neutrophils. In contrast, their elevated NLRs were a result of a low lymphocyte count. There was also a statistically significant difference in the mean platelet count between the two groups, with the lower value being reported in the complicated appendicitis class. This is because large and active platelets are more likely to be sequestered and destroyed at sites of inflammation, leading to a lower platelet count in sepsis and other severe types of inflammatory processes such as ruptured appendicitis [17,18].

Based on the results of this research, NLR, PLR, and MLR may serve as reliable diagnostic tools or adjuncts for identifying cases of acute complicated appendicitis. It was shown that the NLR, PLR, and MLR were all significantly lower in patients who had acute uncomplicated appendicitis. This demonstrated a huge disparity between the two groups in terms of median NLR, PLR, and MLR. Lymphocyte and neutrophil counts were normal, however, in the uncomplicated acute appendicitis group. NLR was shown to be elevated in appendicitis, and this elevation continued as inflammation worsened. This finding agrees with those of past research [19,20]. Despite the apparent correlation between WBC and appendicitis, NLR has been shown to be greater and to have higher diagnostic accuracy than WBC solely [21], and this is reinforced in this study. Appendicitis, like many infectious disorders, causes a rise in neutrophils and a reduction in lymphocytes; hence, NLR may be more precise than the WBC in making a diagnosis of appendicitis [22]. This relative lymphopenia coincides with an increase in neutrophils, which occurs as a physiological reaction to the stress state and, as a consequence, the proportion of these two components to each other may be employed as the biomarker of inflammation during inflammatory responses.

There is some evidence that the monocyte-to-lymphocyte ratio (MLR) can be used to discriminate bacterial from viral infections in febrile individuals [23,24]. Lymphocyte-to-monocyte ratios were not significant between patients with complex and those with uncomplicated appendicitis, according to one retrospective research by Günay et al. [25]. In contrast, individuals with more severe diseases had higher MLR levels in our group (Table 3). Therefore, monocyte-containing ratios like MLR have the potential to be disease-specific and diagnostically useful for complicated appendicitis.

Platelets constitute a crucial connection between inflammation, thrombosis, and atherogenesis [26]. When inflammation occurs, platelets engage with leukocytes and endothelial cells. Platelets, for instance, can promote the adherence and transformation of monocytes and even trigger their development into macrophages by releasing inflammatory chemicals [27]. Platelet release due to fluctuations in the stress hormone levels and temporary lymphopenia must be considered when implementing PLR, with the outcome being an overall increase in PLR under conditions characterized by inflammation and thrombosis [28].

Limitations

Our work has various caveats because of the fact that it is retrospective in nature. The existence of unknown confounding factors that influence our testing components' validity is difficult to determine with certainty. Additionally, our cohort size is rather small and this might have affected the specificity and sensitivity of the test. Another significant drawback is that these individuals may present at a later time after the onset of symptoms, which may have an influence on the NLR.

Although NLR is a readily available and low-cost test, it has to be tested in randomized clinical trials with a broader patient population to see if it can reliably detect acute appendicitis. In order to demonstrate that NLR is preferable to other accessible parameters, it would be advantageous to incorporate additional parameters, such as C-reactive protein (CRP), for comparison with NLR.

## Conclusions

We propose that preoperative NLR is a valuable criterion for distinguishing between uncomplicated and complicated appendicitis, adding that it may be utilized as a companion to the clinical evaluation. Also, this may have ramifications for surgical prioritization, monitoring conservatively treated patients, and patients who do not obtain CT scans frequently including pregnant or pediatric patients.
